# Behavioural Assessment of the A_2a_/NR2B Combination in the Unilateral 6-OHDA-Lesioned Rat Model: A New Method to Examine the Therapeutic Potential of Non-Dopaminergic Drugs

**DOI:** 10.1371/journal.pone.0135949

**Published:** 2015-08-31

**Authors:** Anne Michel, Patrick Downey, Xavier Van Damme, Catherine De Wolf, Rainer Schwarting, Dieter Scheller

**Affiliations:** 1 UCB Biopharma SPRL, Neurosciences TA Biology, Braine l’Alleud, Belgium; 2 UCB Biopharma SPRL, Strategy & Alliance Management, Braine l’Alleud, Belgium; 3 Philipps-University of Marburg, Behavioural Neuroscience, Marburg, Germany; Prince Henry's Institute, AUSTRALIA

## Abstract

In Parkinson’s disease (PD), dopaminergic therapies are often associated with the development of motor complications. Attention has therefore been focused on the use of non-dopaminergic drugs. This study developed a new behavioural method capable of demonstrating the added value of combining adenosinergic and glutamatergic receptor antagonists in unilateral 6-OHDA lesioned rats. Rats were dosed orally with Tozadenant, a selective A_2A_ receptor antagonist, and three different doses of Radiprodil, an NR2B-selective NMDA receptor antagonist. The drugs were given alone or in combination and rats were placed in an open-field for behavioural monitoring. Video recordings were automatically analysed. Five different behaviours were scored: distance traveled, ipsi- and contraversive turns, body position, and space occupancy. The results show that A_2A_ or NR2B receptor antagonists given alone or in combination did not produce enhanced turning as observed with an active dose of L-Dopa/benserazide. Instead the treated rats maintained a straight body position, were able to shift from one direction to the other and occupied a significantly larger space in the arena. The highest “Tozadenant/Radiprodil” dose combination significantly increased all five behavioural parameters recorded compared to rats treated with vehicle or the same doses of the drugs alone. Our data suggest that the A_2A_/NR2B antagonist combination may be able to stimulate motor activity to a similar level as that achieved by L-Dopa but in the absence of the side-effects that are associated with dopaminergic hyperstimulation. If these results translate into the clinic, this combination could represent an alternative symptomatic treatment option for PD.

## Introduction

Despite several decades of active research into novel therapeutic strategies for PD, L-Dopa given in combination with a peripheral dopa-decarboxylase inhibitor, still remains the most powerful drug to alleviate the motor symptoms of Parkinson’s disease [1, 2]. However, the prolonged use of this treatment paradigm also brings considerable side effects. In particular, young patients develop dyskinesia very rapidly after starting L-Dopa therapy, whereas older patients are more prone to cognitive and psychiatric adverse effects [1].

Motor fluctuations and dyskinesia, associated with long-term dopaminergic therapy, can even become more disabling than the disease itself [3] and can significantly impair the quality of life. Besides the post-synaptic adaptations associated to maladaptive striatal plasticity [4, 5], L-dopa-induced dyskinesia (LIDs) largely depend on: (1) the degree of dopamine neuron degeneration; (2) the type of treatment; and (3) the schedule of drug administration [6, 7]. As a consequence, several strategies have been adopted to prevent or avoid the occurrence of L-Dopa-induced dyskinesia (LIDs). These include; delaying the use of dopaminergic treatment [1], modifying the route/dose to ensure continuous dopaminergic drug delivery [8], and the use of the non-selective N-methyl-D-aspartate (NMDA) receptor antagonist amantadine to counteract existing LIDs [9, 10].

Consequently, and in the absence of neuroprotective therapies, the identification of novel non-dopaminergic therapeutic options, which can by-pass the supersensitive dopamine receptors in the lesioned striatum, would be highly desirable. Of particular interest, both adenosine and glutamate receptor antagonists have been shown to modulate motor symptoms, but their effects may be insufficient to induce significant motor activation when given individually as mono-treatment, i.e. without L-Dopa [11–13]. However, preclinical investigations suggest that these non-dopaminergic treatments, i.e. Adenosine A_2A_ (A_2A_) and NMDA receptor subunit NR2B (NR2B) antagonist drugs, offer the great advantage of being devoid of any pro-dyskinetic effects [14], i.e. unlike L-DOPA their use is not expected to induce dyskinesia [15, 16]. Furthermore, NR2B antagonists have not only been shown not to induce dyskinesias but are able to reduce dyskinesia once it has been established both in rats and primates [17, 18].

In a previous report, we demonstrated the enhanced potential of combining A_2A_ and NR2B receptor antagonists in a preclinical rat model of Parkinson’s disease [19]. The unilateral 6-hydroxydopamine (OHDA)-lesioned rat model mimics the status of advanced PD [20] and has been widely used for many years to assess the symptomatic effects of drugs. The massive impairment of the dopaminergic system within one brain hemisphere creates a sensorimotor imbalance between both body sides, leading to specific behavioural deficits. Drugs stimulating postsynaptic receptor sites normally targeted by dopamine (e. g., direct dopamine agonists or L-Dopa) cause the rat to turn in a direction opposite to the damaged side (contraversive rotations). This turning response is considered to be the consequence of dopaminergic D1 and D2 receptor supersensitivity [21], which occurs as a result of the denervation of (parts of) the neostriatum (for a review, see [22]). Supersensitive dopamine receptors are thought to be one of the mechanisms underlying the development of LIDs, so it is worth noting that contraversive rotations as well as measuring the efficacy of dopaminergic drugs may also reflect their propensity to cause involuntary movements.

The phenomenon of drug-induced contraversive turning represents a useful and predictive model to screen for and identify new anti-parkinsonian drugs, and to quantitatively assess them [23, 24]. However, the disadvantage of this model is that it has been entirely tailored for the testing of dopaminergic drugs which induce contraversive rotations by directly stimulating the supersensitive dopamine receptors. Thus, the current experimental paradigm means that this model cannot be used to identify potential novel non-dopaminergic treatments since these would not be expected to induce contralateral rotations! To circumvent this situation, we decided to test the anti-parkinsonian potential of non-dopaminergic drugs in an open-field and to assess behaviours other than rotations.

In our previous report with six different A_2A_/NR2B receptor antagonist combinations, we demonstrated that the level of motor activity (i.e. distance traveled and rearing counts measured in actometers) was significantly higher with combined administrations than with single compounds [19]. In this first study, we visually observed that the automatically quantified locomotor pattern under the A_2A_/NR2B combination did not consist of the typical L-Dopa-like contraversive rotations; instead, this enhanced mobility was characterized by straight displacement in the absence of any trunk torsion.

The objective of the present work was to demonstrate the enhanced therapeutic potential for Parkinson’s disease of combining two drugs, Radiprodil and Tozadenant, on motor activity in hemiparkinsonian rats. Tozadenant is a selective A_2A_ receptor antagonist which has been tested in Phase 2b and has shown clinically relevant and statistically significant effects on “on-time” and an improved score on UPDRS part III [25]. Radiprodil is an NR2B-selective NMDA receptor antagonist which reached Phase 2 for the treatment of neuropathic pain associated with diabetic peripheral neuropathy, but was stopped due to a lack of significant reduction in daily pain scores [26].

In this work, we present a modified experimental design and scoring system to critically assess the improved motor quality observed when the A_2A_/NR2B combination is administered in unilateral 6-OHDA-lesioned rats. We monitored the rats in an open-field with a glass-floor so that we could observe them from below. The subsequent analysis of the video recordings was performed automatically with a video-tracking software. Five relevant behavioural parameters were identified as being suitable for quantitatively evaluating the quality of movement (*distance*; *turning bias*: *ipsi/contra turns*; *body position* and *space occupancy*) on the basis of automatic detection. This technique allowed us to quantify hemilesioned rat motor activity using a set of behaviours representative of normal movements, i.e. similar to those observed in intact rats and, enabled us to discriminate these from the typical contraversive turning response that is observed under L-Dopa treatment.

## Materials and Methods

### 2.1 Drugs

Tozadenant and Radiprodil were obtained from commercial suppliers (Pharmablock and Axon, respectively). Compounds were administered to the animals as suspensions (distilled water containing 5% v/v dimethyl sulfoxide and 1% w/v methyl cellulose; at a volume of 5 ml/kg). L-Dopa methyl ester (Sigma) was dissolved in physiological saline solution at a volume of 5 ml/kg. 6-OHDA-HBr (Sigma) was dissolved at a concentration of 4μg/μl distilled water in 0.02% ascorbic acid. All compounds were freshly prepared before the experiment and homogenized using a magnetic stirrer.

### 2.2 Animals

All animal experiments were performed according to the guidelines of the European Directive 2010/63/EU and Belgian legislation. The ethical committee for animal experimentation from UCB Biopharma SPRL (LA1220040 and LA2220363) approved the experimental protocols. Male Sprague-Dawley rats (Janvier, France) were housed in cages (4 rats per cage) for one week before experimentation. They were kept on a 12:12 light / dark cycle with light on at 06:00 AM and at a temperature maintained at 20–21°C and at humidity of approximately 40%. All animals had free access to standard pellet food and water before assignment to experimental groups. The animals weighed 250–275 g at the time of surgery and 400–450 g at the time of drug testing. Additional enrichment and welfare were provided (Enviro-dri, PharmaServ) before and after the surgery. Animal health was monitored daily by the animal care staff. Surgeries were performed under ketamine and xylazine anesthesia, and all efforts were made to minimize suffering. Sacrifice was done with CO_2_.

### 2.3 6-OHDA lesion

To protect noradrenergic neurons, animals were administered with imipramine HCl (15 mg/kg, ip, Sigma) 15 minutes before surgery. They were subsequently anesthetized with ketamine (Ceva, 75 mg/kg) and xylazine (Bayer, 10 mg/kg) and placed into a stereotaxic frame (David Kopf Instrument). 6-OHDA was injected into the right ascending medial forebrain bundle at the following coordinates (in mm) relative to bregma and surface of the dura, AP = -3.5, ML = -1.5, DV = -8.7 [27]. Each rat received one injection of 6-OHDA (4 μg/μl) over a period of 5 minutes (0.5 μl/min) for a total of 10 μg per rat. Animals were monitored for 3 weeks to ensure full recovery and habituation to the environment and experimenters. On day 21 after surgery, all rats were challenged with a small subcutaneous dose of apomorphine (Sigma, 0.05 mg/kg). Rats showing more than 90 contraversive rotations (360°) over a 45-minute recording period were included in the study. It has been previously demonstrated that rats meeting this criterion have a unilateral loss of dopaminergic neurons and a unilateral depletion of striatal dopamine of over 95% [19].

### 2.4 Behavioural assessment

The specific behavioural profile of unilateral 6-OHDA-lesioned rats was recorded via a video acquisition system which allowed the simultaneous analysis of eight rats. Rats under treatment were placed into the open-field (50 x 40 cm) and behaviour was video-recorded for 55 minutes. The rats were filmed from below. Each of the 8 clear acrylic chambers was placed on a clear glass bottom, held by a robust frame. A removable acrylic plate served as a lid. A portable digital camera (Samsung, SCB-3001 PH) was positioned directly underneath each arena in order to view the whole surface covered by the chamber. Lighting was provided by neon-tubes fixed singularly on each leg of the frame. Video sequences were recorded on hard disk for storage and subsequently transferred to PC for analysis. After the recordings, the videos were subsequently analyzed by the Ethovision program (Noldus, version 9.0). The animal was defined according to three points (nose, center and tail) which referred to three coordinates. The rat’s movements, its body position and the space occupied, were set-up according to the filter analysis defined with specific criteria and thresholds in the program. These set-ups allowed the definition of specific behavioural parameters with the Ethovision system. Only the parameters that did not show any significant statistical correlation between them after treatment with dopaminergic and non-dopaminergic drugs were selected for the current experiment and future analyses.

The parameters labeled as “Distance”, “Counter Clockwise rotations (CCW)”, “Clockwise rotations (CW)”, “Body Elongation—Stretched” were extracted from the Ethovision analysis. One additional parameter, referring to the gyration radius exhibited by the rats under drug treatment (for definition, see S1 Fig) which was defined as the “space occupancy” was calculated from the raw data files (animal position as a function of time), exported from the system and processed in an Excel macro with time bins of ten seconds. To facilitate the behavioral description in the text, these behavioral parameters were specifically labeled and defined as followed:

- *Distance*: the total distance traveled by the animal (cm).
- *Ipsi Turns (CCW)*: the number of 360° turns in the same direction as that of the lesion. The “Minimal Distance Moved” was set to 1cm.
- *Contra Turns (CW)*: the number of 360° turns in the opposite direction to that of the lesion. The “Minimal Distance Moved” was set to 1cm.
- *Body position* (Body Elongation-stretched): discrimination of both intensity and frequency of the body position; measurement of the time spent in a position wherein the rat stands on its four paws (quadrupedal posture) without any bent position of the trunk. The threshold was fixed at 70%.
- *Space occupancy* (gyration radius): for every 10-sec interval, calculation of the average distance between each animal position (*25 xy/sec*) minus the average position of the animal within this specific 10-sec interval. The mean of these distances is calculated for the period of observation (3300 seconds). This parameter reflects the ability of the rats to use the entire space of the arena and is measured in centimeters (S1 Fig).

### 2.5 Experimental design

Drug-naïve 6-OHDA rats were orally administered with vehicle (VEH), Radiprodil at 1, 2 or 3 mg/kg (RAD), Tozadenant 30 mg/kg (TOZ), or the combination of Radiprodil + Tozadenant 30 mg/kg (RAD/TOZ) and were then placed into the open-field 60 minutes after the drug injection. After a 5-min habituation phase, behaviour was video-recorded for 55 minutes. Three different doses of Radiprodil (1, 2 and 3 mg/kg, po) were combined to vehicle or to a fixed dose of Tozadenant (30 mg/kg, po). For comparison, two additional groups (drug-naïve lesioned rats) were tested within the same system for assessing the effect of L-DOPA treatment. The first group received L-Dopa 14 mg/kg plus benserazide 3.5 mg/kg (ip), and the second group was treated with L-Dopa 25 mg/kg (ip) without dopa-decarboxylase inhibitor. The combined administration of L-Dopa plus benserazide was considered as the high and long lasting dopaminergic stimulation whereas the administration of a 25mg/kg dose of L-Dopa without dopa-decarboxylase inhibition was considered the weak and short active stimulation (S2 Fig).

### 2.6 Statistical analysis

The impact of the treatment on the five different behaviours measured in the open-field was analyzed with two-way analysis of variance (ANOVA) followed by subsequent post hoc test. Statistical analyses were performed separately on the five different behavioural measures. Each two-way ANOVA incorporated the dose of Tozadenant (2 levels: vehicle or 30 mg/kg), the dose of Radiprodil (4 levels: vehicle, 1, 2 or 3 mg/kg) as between-group factors. The statistical interaction between the two drugs was explored in order to assess if the two drugs interacted according to an additive (i.e. non-significant interaction between Tozadenant and Radiprodil) or a synergistic mode (i.e. significant interaction between Tozadenant and Radiprodil).

In case of the absence of variance homogeneity (Levene’s test for equal variances), square-root or logarithmic transformations were conducted. The selection of the transformation was based on Cox-Box test and lambda calculation. Data were expressed as means +/- standard error of the mean and every pharmacological condition was tested with 8 rats. The multiple pairwise comparisons among the eight different means were performed by Tukey’s post hoc test.

The comparisons between the two different administrations of L-Dopa (L-Dopa/benserazide versus L-Dopa 25 mg/kg) were done with Student’s *t*-tests.

Statistical analyses were performed using the Statistica software (StatSoft Inc., OK, USA). Statistical significance was assumed if p values were less than 0.05.

## Results

### 3.1 Distance traveled

**Fig 1:**
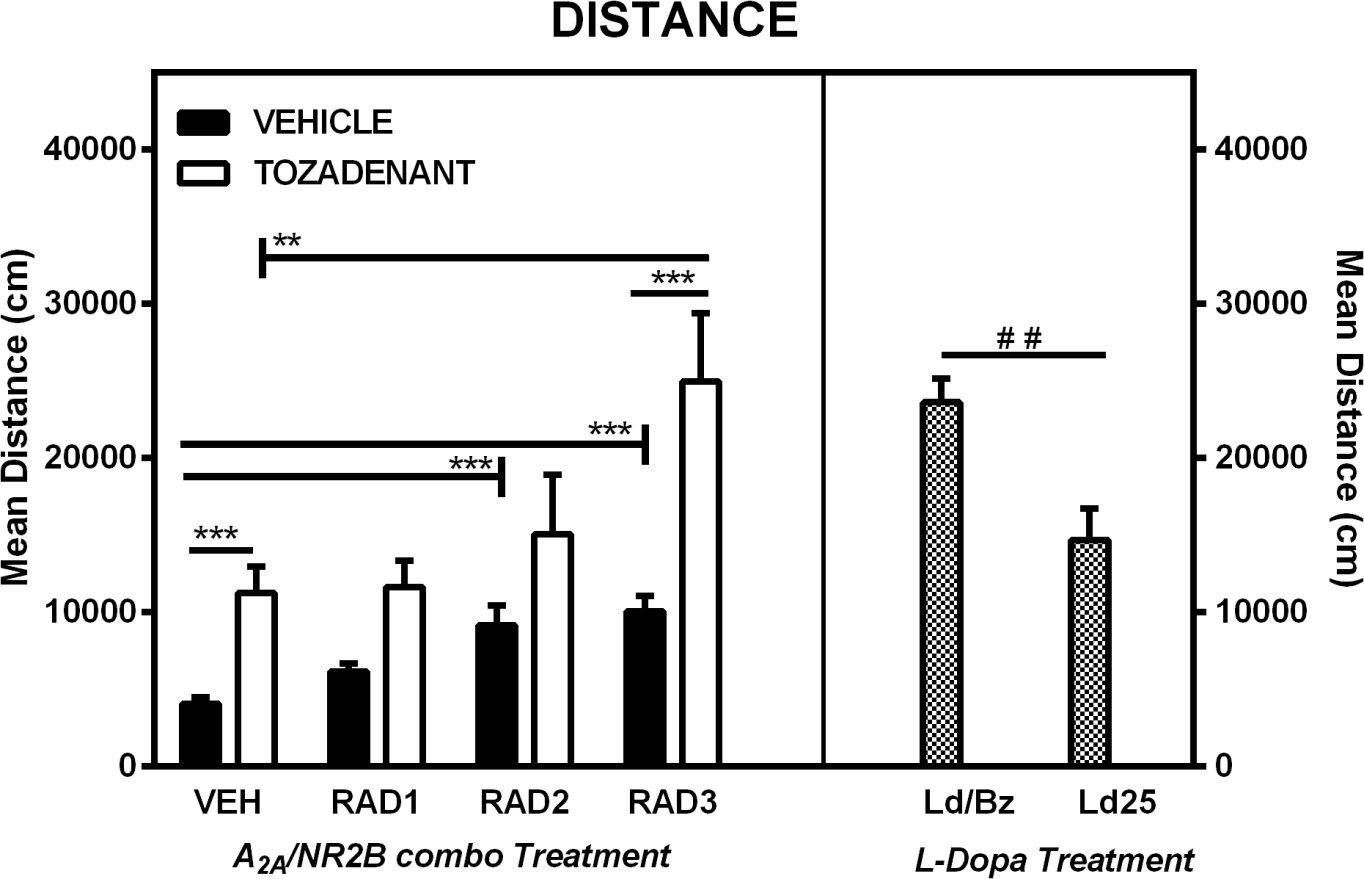
*Distance* traveled (Ethovision, Noldus) in an open-field of 6-OHDA lesioned rats. Comparison of the effect of three different doses of Radiprodil (1, 2 or 3 mg/kg) given in combination with a fixed dose of Tozadenant (30 mg/kg). For comparison, a dose of L-Dopa 14 mg/kg plus benserazide 3.5 mg/kg and a dose of L-Dopa 25 mg/kg (without benserazide) were also tested. RAD3/TOZ is significantly higher than TOZ and RAD3 (**, p<0.01, ***, p<0.001). VEH is significanly lower than RAD2, RAD3 and TOZ (***,p<0.001). L-Dopa/Benserazide is significantly higher than L-Dopa 25 mg/kg (^##^, p<0.01).

Using the Ethovision software, we reproduced the effect we had already observed with automated photobeam activity chambers: an increase in the distance traveled in 6-OHDA rats receiving the combination of A_2A_/NR2B antagonist drug in comparison to rats having received either drug alone. This effect was observed with the combination of Radiprodil 3 mg/kg and Tozadenant 30 mg/kg (RAD3/TOZ) but not when lower doses of Radiprodil were combined with Tozadenant (Fig 1). These observations were supported by two-way ANOVA which showed a significant effect of Tozadenant [F(1,87) = 55.91, p<0.001], a significant effect of Radiprodil [F(3,87) = 19.56, p<0.001], and a significant Tozadenant x Radiprodil interaction [F(3,87) = 2.75, p<0.05]. Additional post hoc tests showed that the group treated with RAD3/TOZ demonstrated significantly higher levels of distance traveled than the group treated with Tozadenant or with Radiprodil 3 mg/kg alone (p<0.01, p<0.001). These results strongly suggest the presence of a synergistic effect between Radiprodil and Tozadenant for the parameter “distance traveled”. The post hoc analysis also demonstrated that rats treated with Tozadenant 30 mg/kg, Radiprodil 2 or 3 mg/kg had significantly higher levels of distance traveled than the vehicle-treated rats (p<0.001).

Interestingly, the RAD3/TOZ group also showed level of distance traveled that were comparable to the the group treated with L-Dopa/benserazide (Student’s *t*-test, t = -0.28 with p = 0.78). By contrast, significantly higher levels of distance traveled were found in the group treated with L-Dopa and benserazide compared to those treated with L-Dopa (25 mg/kg) alone (Student’s *t*-test, p<0.01).

### 3.2 Turning bias

**Fig 2:**
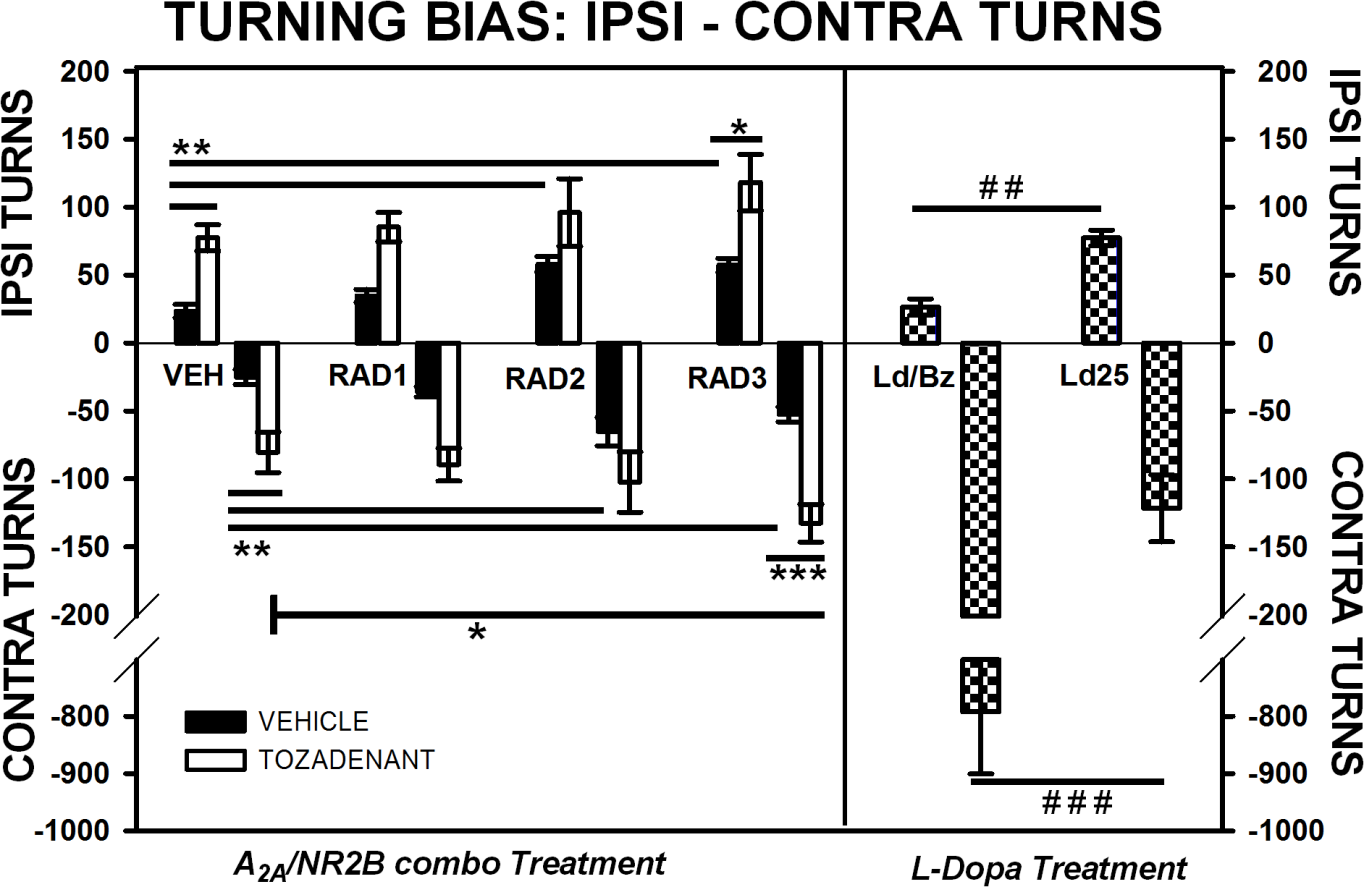
*Ipsi- and contra turning bias* (Ethovision, Noldus) in open-field of 6-OHDA rats. Comparison of the effect of three different doses of Radiprodil (1, 2 or 3 mg/kg) given in combination with a fixed dose of Tozadenant (30 mg/kg). For comparison, a dose of L-Dopa 14 mg/kg plus benserazide 3.5 mg/kg and a dose of L-Dopa 25 mg/kg (without benserazide) were also tested. *Ipsi turns*: RAD3/TOZ is significantly higher than RAD3 (*, p<0.05). VEH significantly lower than TOZ, RAD2 and RAD3 (**, p<0.01); L-Dopa 25 mg/kg is significantly higher than L-Dopa/Benserazide (^##,^ p<0.01). *Contra turns*: RAD3/TOZ is significantly higher than TOZ (*, p<0.05) and RAD3 (***,p<0.001). VEH is significantly lower than TOZ, RAD2 and RAD3 (**, p<0.01). L-Dopa/benzerazide is significantly higher than L-Dopa 25 mg/kg (^###^, p<0.001).

Typically, rats treated with L-Dopa and benserazide showed profound turning towards the contralateral side. This group of rats showed significantly higher levels of contraversive turns (p<0.001, Student’s *t*-test) in comparison to rats treated with L-Dopa alone (25 mg/kg). This increase in the L-Dopa/benserazide group was at the expense of the level of ipsi turns. Accordingly, rats treated with L-Dopa 25 mg/kg showed significantly superior levels of ipsilateral turns than those treated L-Dopa/benserazide (p<0.01) (Fig 2).

Rats treated with Tozadenant, Radiprodil or the Radiprodil/Tozadenant combination did not show any turning bias: all groups were able to turn towards both sides. However, only rats treated with the RAD3/TOZ combination showed significantly more contra turns than those treated with the compounds alone at the same dose. Two-way ANOVA and post hoc analysis on contra turns supported this observation and showed a significant effect of Tozadenant [F(1,87) = 60.81, p<0.001], Radiprodil [F(3,87) = 10.09, p<0.001], but no “Tozadenant x Radiprodil” for the level of contra turns. Tukey post hoc comparisons showed that (1) only the RAD3/TOZ combination increased the level of contralateral turns in comparison to the same dose of the drugs alone (RAD3, p<0.001, TOZ, p<0.05);(2) Tozadenant alone, Radiprodil 2 and Radiprodil 3mg/kg significantly increased the levels of contralateral turns in comparison to vehicle-treated subjects (p<0.01);

For ipsi turns, two-way ANOVA showed a significant effect of Tozadenant [F(1,87) = 58.54, p<0.001] and Radiprodil [F(3,87) = 9.79, p<0.001], and no interaction between the drugs. Tukey post hoc tests specified that rats treated with Tozadenant, Radiprodil 2 and Radiprodil 3 mg/kg had significantly higher levels of ipsilateral turns than vehicle-treated rats (p<0.01). However, rats treated with the combined RAD3/TOZ had higher level of ipsi turns than rats treated with Radiprodil 3 (p<0.05) but not than rats treated with Tozadenant 30 mg/kg.

### 3.3 Body position

**Fig 3:**
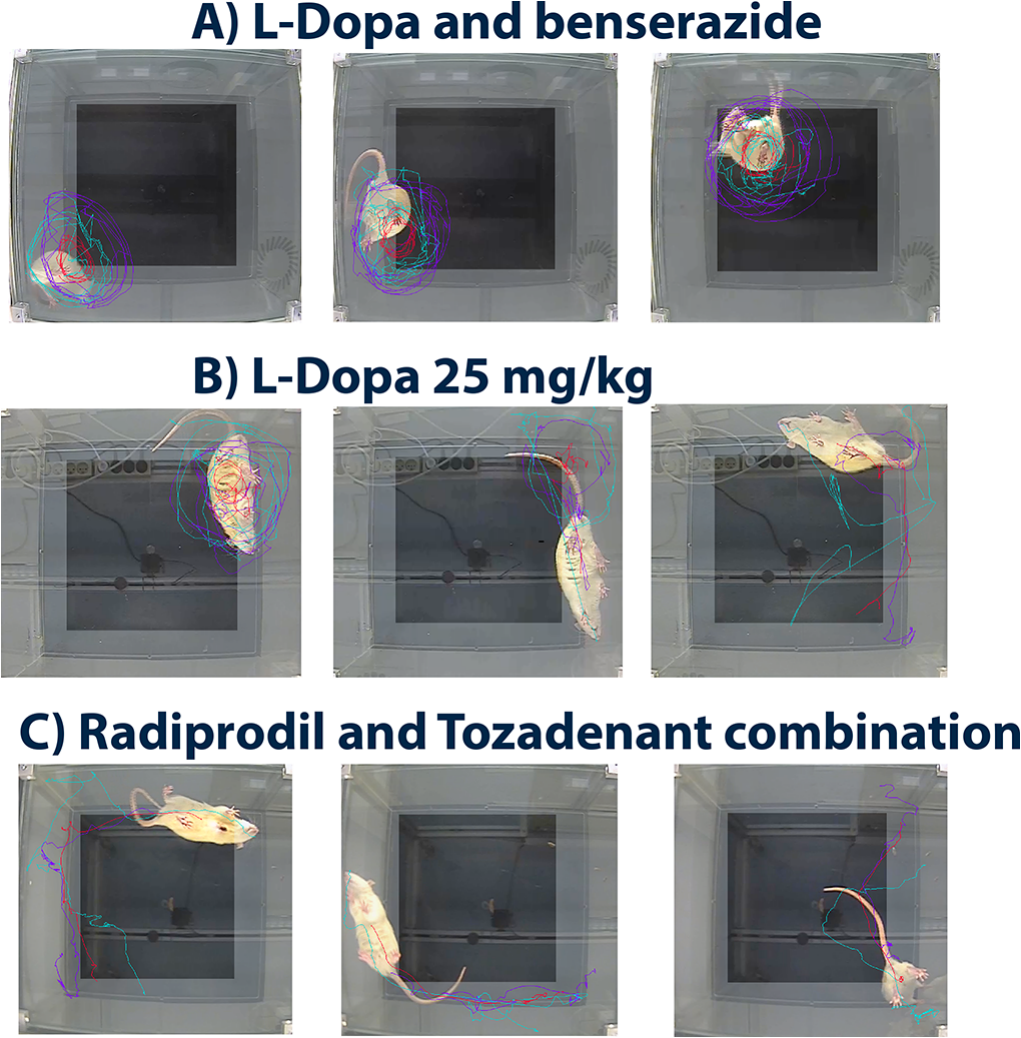
Comparison of the behavioural profile (*space occupancy* and *body position*) of 6-OHDA-lesioned rats after acute administration of (A) L-Dopa 14 mg/kg/benserazide 3.5 mg/kg; (B) L-Dopa 25 mg/kg and, (C) RAD3/TOZ combination. Fig 3A: Typically, lesioned rats even after the first administration of L-Dopa/benserazide showed a bent body position with some loss of floor contact with forepaw(s) during rotational activity. The contraversive rotations are characterized by a small diameter of the circle drawn when the nose turns around the center of gravity. The trajectory refers to a sum of circles express in various locations of the surface arena; Fig 3B: Rats treated with a weak dose of L-Dopa (25 mg/kg) expressed a mix of contralateral rotations and straight movement during exploration of the floor. The body position is straight and all the paws are in contact with the floor during the rotations; Fig 3C Rats treated with RAD3/TOZ combination showed a straight body position with all paws in contact with the floor while explorating. The trajectories are straight and space occupancy is large; there are some alterance during exploration of the walls and the center of the arena.

**Fig 4:**
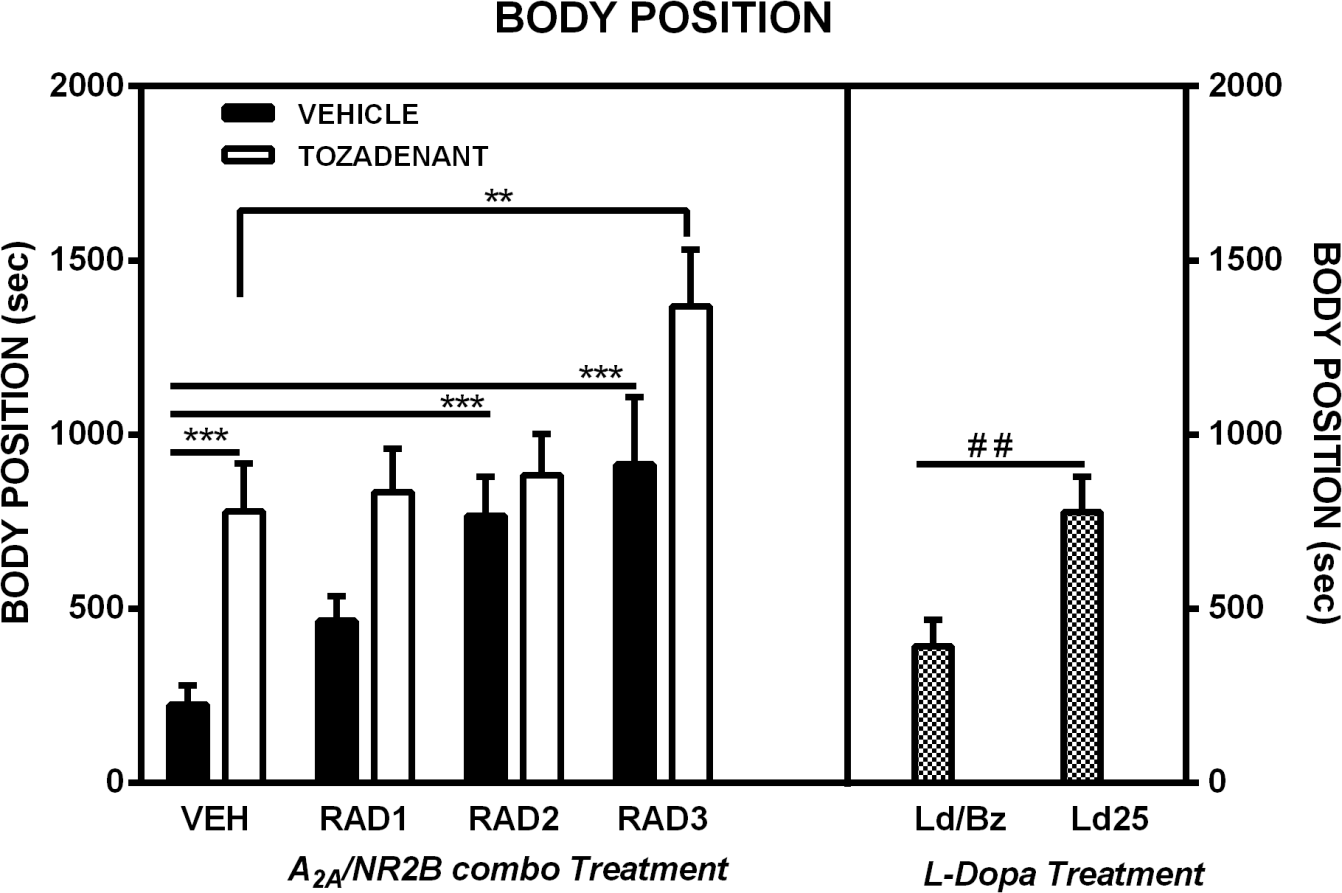
*Body position* in open-field of 6-OHDA rats (Ethovision, Noldus). Comparison of the effect of three different doses of Radiprodil (1, 2 or 3 mg/kg) given in combination with a fixed dose of Tozadenant (30 mg/kg). For comparison, a dose of L-Dopa 14 mg/kg plus benserazide 3.5 mg/kg and a dose of L-Dopa 25 mg/kg (without benserazide) were also tested. RAD3/TOZ is significantly higher than VEH and TOZ (**, p<0.01). VEH is significantly lower than TOZ, RAD2 and RAD3 (***, p<0.001). L-Dopa 25 mg/kg is significantly higher than L-Dopa/Benserazide (^##^, p<0.01).

Typically, when 6-OHDA-lesioned rats are administered with a fully active dose of dopaminergic drugs, they exhibit a bent body position during their rotational activity together with some loss of contact with the floor with one or both forelimbs. These behavioural effects were observed after the acute L-Dopa/benserazide administration (Fig 3A). By contrast, rats receiving the lower dose of L-Dopa showed a straighter position of the trunk during rotational activity with adequate and full contact of the four paws on the floor (Fig 3B). Statistical analysis showed that rats treated with L-dopa 25 mg/kg spent larger amounts of time on their four paws without any bent position of the trunk in comparison to rats treated with L-Dopa and benserazide (Student’s *t*-test, p<0.01) (Fig 4). Typically, the non-dopaminergic drugs did not induce any trunk torsion in unilateral 6-OHDA-lesioned rats and showed very good restoration of the body position in comparison to vehicle-treated rats (Fig 3C). This observation was supported by statistical analysis which showed significant effects of Radiprodil [F(3,87) = 17.45, p<0.001], Tozadenant [F(1,87) = 26.30, p<0.001], and a “Tozadenant x Radiprodil” interaction [F(3,87) = 3.36, p<0.05]. In addition, the rats treated with the RAD3/TOZ combination spent more time in this position than rats treated with vehicle or with Tozadenant alone but not Radiprodil (Tukey post hoc test, p<0.01). Tozadenant, Radiprodil 2 and Radiprodil 3 mg/kg also restored the quality of the body position of hemiparkinsonian rats. Post hoc comparisons showed that these groups of rats spent significantly more time in the quadrupedal and non-bent body position than the vehicle-treated rats (Tukey post hoc test, p<0.001).

### 3.4 Space occupancy

The direct visual observation showed that, despite a high level of motor stimulation, rats under the RAD3/TOZ combination recovered their capacity to explore the environment by inspecting the whole surface of the testing arena (Fig 3C). They did not demonstrate any “fixed and stereotyped biased” motor program (contraversive turns), as observed when 6-OHDA-lesioned rats are higly stimulated with dopaminergic drugs (Fig 3A). Interestingly, rats treated with L-Dopa 25 mg/kg showed the ability to shift from the stereotypical rotational behaviour to straight displacement in order to cover larger surface during exploration (Fig 3B).

**Fig 5:**
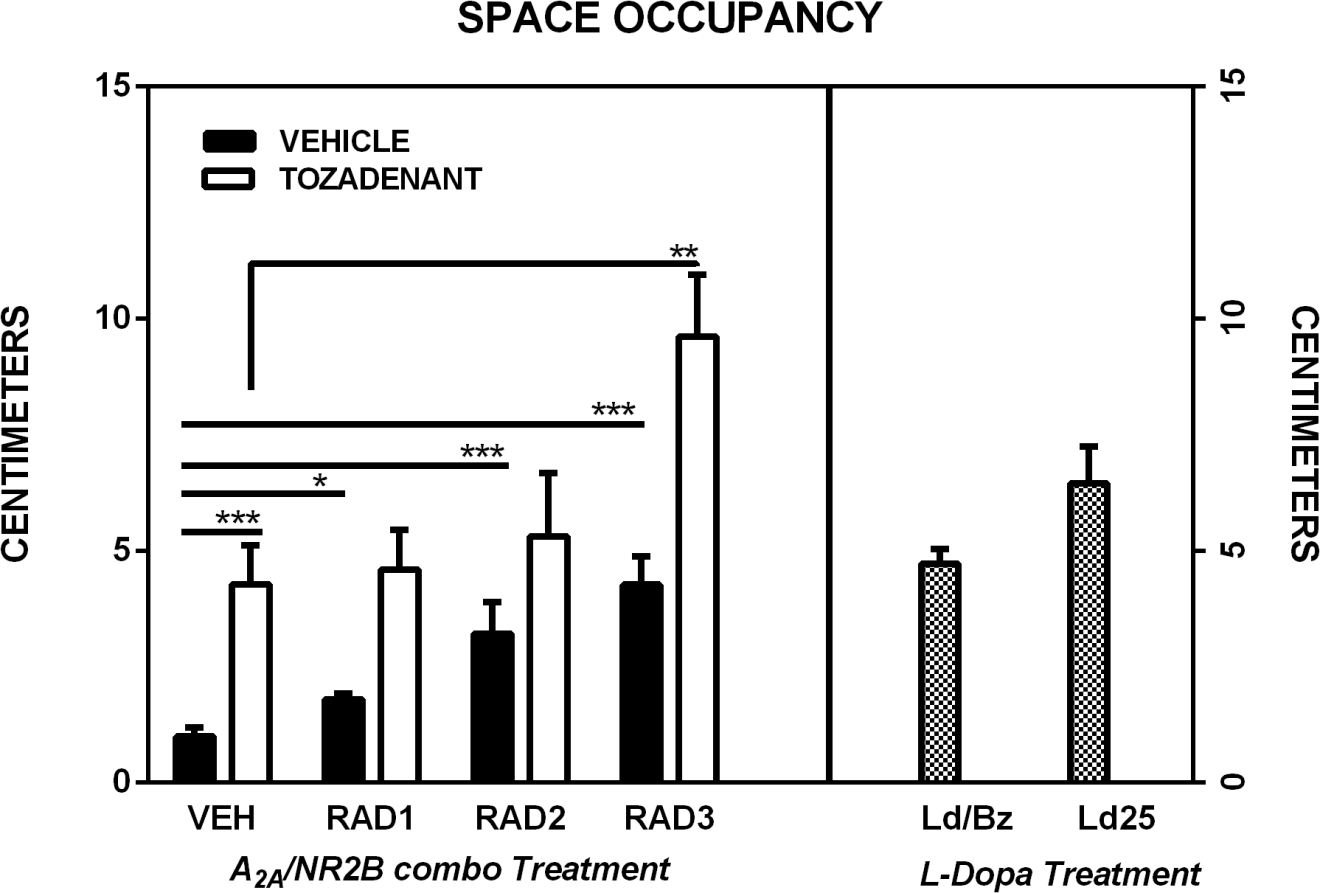
*Space occupancy* in open-field of 6-OHDA rats (Ethovision, Noldus). Comparison of the effect of three different doses of Radiprodil (1, 2 or 3 mg/kg) given in combination with a fixed dose of Tozadenant (30 mg/kg). For comparison, a dose of L-Dopa 14 mg/kg plus benserazide 3.5 mg/kg and a dose of L-Dopa 25 mg/kg (without benserazide) were also tested. RAD3/TOZ is significantly higher than TOZ (**,p<0.01). VEH is significantly lower than RAD 1 (*, p<0.05), TOZ, RAD2 and RAD3 (***, p<0.001).

Unilaterally 6-OHDA-lesioned rats treated with the RAD3/TOZ combined administration showed significant enlargement of the trajectory while moving in comparison to the groups treated with vehicle or treated with the same dose of the drug given alone (Fig 5). This observation was supported by two-way ANOVA which showed significant effects of Tozadenant [F(1,87) = 50.00, p<0.001], Radiprodil [F(3,87) = 22.00, p<0.001], and a significant Tozadenant x Radiprodil interaction [F(3,87) = 4.22, p<0.01]. Additional post hoc comparisons showed that rats treated with the RAD3/TOZ combination occupied larger space than the rats treated with vehicle (p<0.001) and Tozadenant alone (p<0.01). Also, the groups treated with Tozadenant (p<0.001), Radiprodil 1 (p<0.05), 2 and 3 mg/kg (p<0.001) showed significant enlargements of the trajectory in comparison to vehicle-treated rats (Tukey post hoc test).

## Discussion

A new method has been developed enabling a more detailed, but quantitative analysis of different components of the locomotor activities of rats. That development was necessary as the motor effects under a non-dopaminergic treatment do not necessarily reproduce the typical dopaminergic responses (rotations) in unilaterally 6-OHDA-lesioned rats.

In a previous report, we demonstrated that the combined administration of an A_2A_ with an NR2B receptor antagonist drug significantly increased the levels of locomotor activity in unilateral 6-OHDA-lesioned rats when compared to the effect with the drugs when given alone [19]. However, while we observed that the motor activation resulting from the combined drug administration was entirely different from the typical contraversive rotations produced by dopaminergic drugs, the existing tools made it difficult to quantify the apparent increases in motor quality that were associated with combination treatment. In this work, using large field apparatus and a sophisticated monitoring approach, we were able to quantify increases in the quality of movement. This was achieved by measuring specific parameters, namely, the ability to perform straight displacements over a large trajectory and space occupancy while being able to move towards both the ipsi- and contraversive sides and maintaining a quadrupedal body position.

In order to quantify the behavioural improvement visually observed under the combined A_2A_/NR2B administration and to compare it to a dopaminergic stimulation (L-Dopa and benserazide versus L-Dopa alone), we monitored the unilateral 6-OHDA-lesioned rats from below in order to be able to capture their body shapes, paw placements, and to monitor the tracks of their locomotion. These latter parameters gave information on the quality of the body position (bent or straight) and on the locomotor trajectories (small circles or large walk) as well as on paw use (quadrupedal posture).

Regarding those behavioral observations under the A_2A_/NR2B combination, several topics can be discussed. First, we reproduced with this new system the results previously observed in activity photo beam cages [19]: i.e a significant increase of the distance traveled with the A_2A_/NR2B combination in comparison to rats treated with the same dose of the drugs alone. However, such effects were only significant when the dose of Radiprodil, given in combination with Tozadenant, was 3 mg/kg. Interestingly, we also observed that the distance measured with the highest Radiprodil dose combination was in the same range as that observed with the high dose of L-Dopa and benserazide (Fig 1). Such results indicate that the Radiprodil 3 and Tozadenant 30 mg/kg combination stimulated the rats to a similar degree as that achieved by a high dose of L-Dopa/benserazide. We also observed that Tozadenant 30 mg/kg, Radiprodil 2 and 3 mg/kg, when given alone, were able to significantly increase the level of distance traveled in 6-OHDA-lesioned rats in comparison to vehicle-treated subjects. However such activity while statistically significant, was rather weak.

Secondly, we observed that rats treated with the A_2A_ and/or NR2B antagonist drugs were able to shift during locomotion from the right to the left and vice versa; this was clearly demonstrated with the levels of ipsi- and contra turns being equivalent for both directions and also, those turns were entirely different from the stereotyped L-Dopa-induced contraversive turns. Such an ability to shift from one direction to the other was also noticeble with rats treated with the low dose of L-Dopa (25 mg/kg without benserazide) but not with rats treated with L-Dopa plus benserazide (Figs 2 and 3). This latter group demonstrated very high levels of contraversive turns which confirmed the high stimulation of post-synaptic dopamine receptors of the lesioned side. This second observation puts emphasis on the artificial aspects of the “L-Dopa-induced contraversive rotations” test for measuring the anti-parkinsonian efficacy of compounds. This observation further supports the notion that the recording of the contraversive rotations induced by dopaminergic drugs in unilaterally 6-OHDA-lesioned rats may not merely reflect the anti-parkinsonian activity of those drugs. Indeed, it has been suggested that the robust contraversive rotations produced by dopaminergic stimulation may in fact show the inherent ability of drugs to induce motor complications[28], with such abnormal hyperactivity being far from any normal behaviour [29]. Interestingly, Tozadenant at 30 mg/kg and Radiprodil at 2 and 3 mg/kg increased the levels of ipsi- and contra turns recorded in comparison to vehicle-treated rats. Despite significant effect, these latter were weak but nevertheless in agreement with studies showing ability of these two drug classes to produce some rotations, and consequently some motor activation, when given in monotherapy in lesioned animals [15, 30, 31]. Unfortunately, only the contraversive rotations have been reported, supposedly due to bias induced by rotometers. However, we will see below that the motor stimulation achieved by the non-dopaminergic drugs used here, is qualitatively entirely different from that observed with L-Dopa.

Thirdly, we observed that the “body position”, i.e. the ability of the unilaterally lesioned rats to spend time in a horizontal quadrupedal and non-bent position, was improved in comparison to vehicle- and L-Dopa/benserazide-treated rats for the groups treated with Tozadenant, or Radiprodil 2 or 3 mg/kg. These rats recovered some mobility without showing the typical bent position observed with dopaminergic agents. This observation reinforces previous observations which suggest that A_2A_ antagonist drugs are not pro-dyskinetic when given alone and is in line with a report showing that A_2A_ receptor antagonists do not induce dyskinesias in drug-naïve rats [14]. In addition, the rats treated with the combination of Radiprodil 3 and Tozadenant 30 mg/kg spent significantly more time in this position when compared to rats treated with the same dose of the drugs alone. Interestingly, rats that received the low dose of L-Dopa (25 mg/kg without benserazide) showed a significant improvement of the body position in comparison to rats treated with L-Dopa plus benserazide.

Fourthly, we demonstrated that the diameter of the trajectory observed with rats treated with the highest dose of combined A_2A_/NR2B administration was enlarged when compared to groups treated with the same dose of each drug alone. Also, the space occupancy was larger for rats treated with the low dose of L-Dopa in comparison to those treated with L-Dopa and benserazide. This measure demonstrates that rats receiving a low dopaminergic stimulation or the A_2A_/NR2B combination were able to explore the entire surface of the testing arena whereas this was not the case for rats which received high dopaminergic stimulation.

These two last parameters (i.e. *body position* and *space occupancy*) demonstrated that rats treated with a drug inducing high postsynaptic dopaminergic stimulation, were in a fixed and stereotyped motor program. This hyperactivity was expressed through a high level of tight contraversive rotations which prevented them from exploring the surrounding environment. By contrast, the current newly developed experimental paradigm was able to detect and demonstrate the potential efficacy of non-dopaminergic drugs to restore significantly higher levels of normal locomotor activity: restoration of behavioural quadrupedal straight body position, the ability to shift from one direction to the other while exploring the environment, and the absence of any stereotyped rotations. In addition, our statistical analysis demonstrated that for some behavioural parameters, the two drugs interacted according to a synergistic mode (i.e. significant “Radiprodil x Tozadenant” interaction). This effect was observed for distance, body position, and space occupancy. Whereas for the turning bias (contra- and ipsiversive turns) both drugs interacted according to an additive mode (non significant “Radiprodil x Tozadenant” statistical interaction). These results show that there are subtle interactions between these two drug classes and, depending on the behavioral parameters analysed, some additive or synergistic effects may be found. This observation reinforces the need to quantify multiple behavioural parameters in order to get a more complete picture of how drugs affect rodent behaviours. Reducing the complex pharmacological action of drugs to one behavioural parameter is fraught with risk and may lead to misinterpretation of the results. In order to validate this new behavioural method further, it would be good to test the positive effects of the A_2A_/NR2B combination in the bilaterally lesioned primate model. In this model, one could assess whether the effects observed in unilaterally lesioned rats are also observed when dopaminergic degeneration is bilateral and also determine if effects seen in rodents are translatable to primates which have a much larger behavioural repertoire.

In summary, in this study we show the development of a new method to analyse the positive effects of non-dopaminergic pharmacological treatment on the behaviour of unilaterally 6-OHDA-lesioned rats. Using this new experimental paradigm we demonstate that the combined administration of Tozadenant (A_2A_ receptor antagonist) with Radiprodil (NR2B receptor antagonist) significantly improves both the quantity and quality of the locomotor activity in comparison to L-Dopa/benserazide treatment. Typically, the two non-dopaminergic drugs, when administered alone, marginally increase the level of motor activity but preserve normal-like rodent behaviours. However, when combined together, the level of motor activity significantly increases in comparison to the drugs alone and this is not at the expense of the quality of the behaviour which remains almost normal although the rodents are unilaterally lesioned. If these results can be further validated in a primate model of Parkinson’s disease, it would make a strong case for considering the combined administration of an A_2A_ and an NR2B antagonist for the symptomatic treatment of Parkinsonian patients.

## Supporting Information

S1 Fig Graphical representation of the animal positions during a 10-sec interval. The coordinates of the center of the circle represent the coordinates of the average position of the animal within a specific time interval. The radius of the circle corresponds to the “gyration radius” and refers to the space occupancy of the rat within the testing arena and is measured in cm.
(TIF)

S2 Fig Duration of the effects with two doses of L-Dopa on the level of contraversive rotations in unilateral 6-OHDA-lesioned rats. Comparison of the effects observed after dosing with a combined administration of L-Dopa 14 mg/kg plus benserazide 3.5 mg/kg versus L-Dopa 25 mg/kg without benserazide (n = 8 rats/group).
(TIF)

S1 Video Effect of an acute injection of 25 mg/kg of L-Dopa (ip) on the behavioural profile in a unilaterally 6-OHDA-lesioned rat. (WMV)

S2 Video Effect of an acute injection of L-Dopa 14 mg/kg plus benserazide 3.5 mg/kg (ip) on the behavioural profile in a unilaterally 6-OHDA-lesioned rat. (WMV)

S3 Video Effect of an acute injection of the combination of Radiprodil 3 mg/kg and Tozadenant 30 mg/kg (ip) on the behavioural profile in a unilaterally 6-OHDA-lesioned rat. (WMV)

S4 Video Effect of an acute injection of Radiprodil 3 mg/kg (ip) on the behavioural profile in a unilaterally 6-OHDA-lesioned rat. (WMV)

S5 Video Effect of an acute injection of Tozadenant 30 mg/kg (ip) on the behavioural profile in a unilaterally 6-OHDA-lesioned rat. (WMV)
