# Supplementary figures and images for: Behavioural Assessment of the A2a/NR2B Combination in the Unilateral 6-OHDA-Lesioned Rat Model: A New Method to Examine the Therapeutic Potential of Non-Dopaminergic Drugs
Source: PLoS One. 2015 Aug 31;10(8):e0135949. doi: 10.1371/journal.pone.0135949 (PMC4555651; doi:10.1371/journal.pone.0135949)

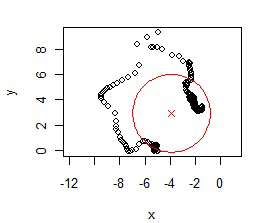

Supplement: S1 Fig — The coordinates of the center of the circle represent the coordinates of the average position of the animal within a specific time interval. The radius of the circle corresponds to the “gyration radius” and refers to the space occupancy of the rat within the testing arena and is measured in cm. (TIF) [file pone.0135949.s001.tif]

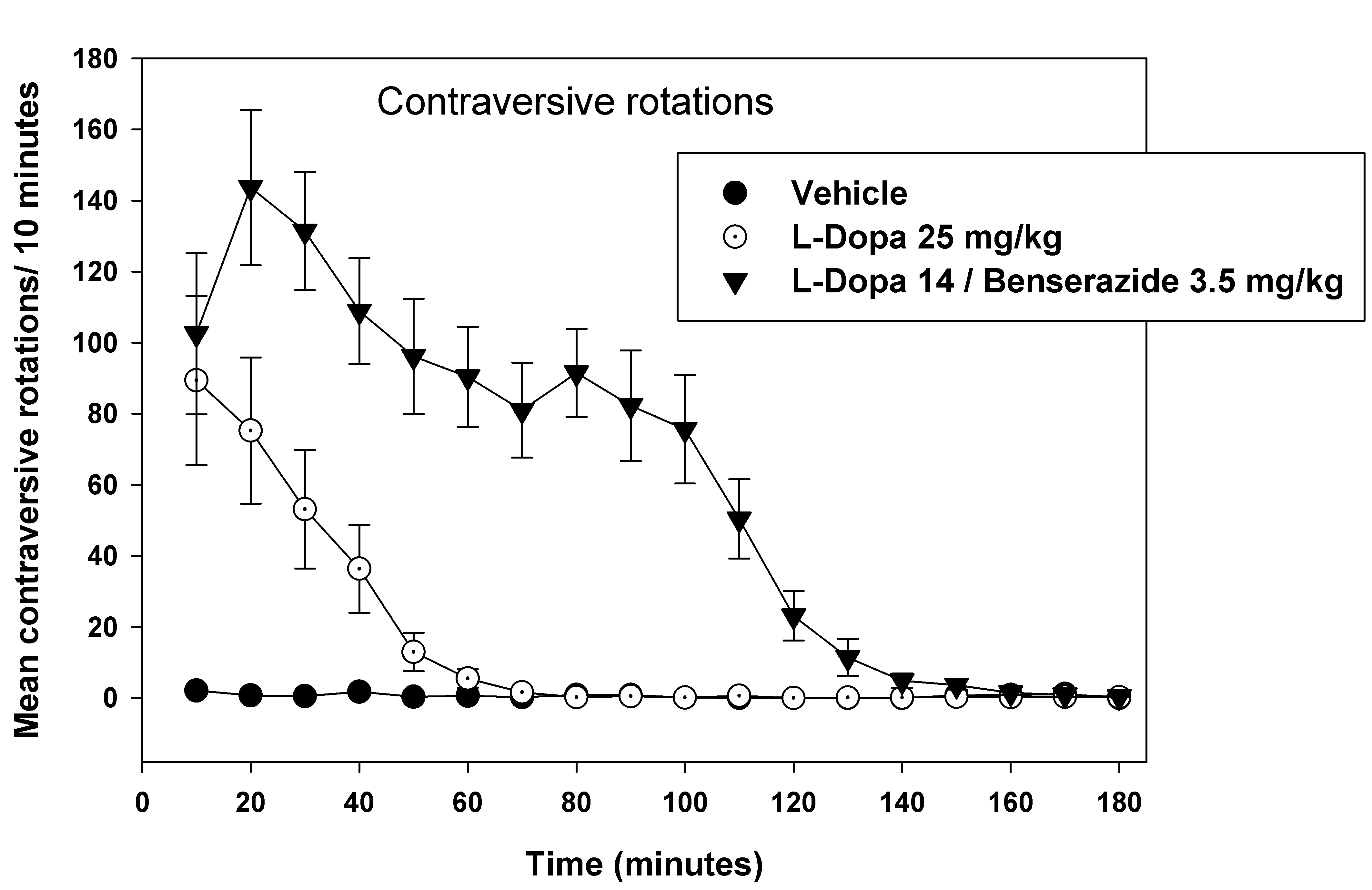

Supplement: S2 Fig — Comparison of the effects observed after dosing with a combined administration of L-Dopa 14 mg/kg plus benserazide 3.5 mg/kg versus L-Dopa 25 mg/kg without benserazide (n = 8 rats/group). (TIF) [file pone.0135949.s002.tif]
